# Reproductive Genetic Carrier Screening in Romania: A Couple-Based Study of Pathogenic Molecular Variants

**DOI:** 10.3390/ijms27083581

**Published:** 2026-04-17

**Authors:** Miruna Gug, Cristina Gug, Aurora Alexandra Jurca, Tudor-Alexandru Popoiu, Raul Patrascu, Paula Andreea Roman, Larisa Olteanu, Nicoleta Andreescu

**Affiliations:** 1 Doctoral School of Medicine, “Victor Babes” University of Medicine and Pharmacy, 300041 Timisoara, Romania; miruna.gug@umft.ro (M.G.); tudor.popoiu@umft.ro (T.-A.P.); 2 Medical Genetics, Department of Microscopic Morphology, Faculty of Medicine, “Victor Babes” University of Medicine and Pharmacy, 300041 Timisoara, Romania; andreescu.nicoleta@umft.ro; 3 Medical Genetics Office Doctor Gug, 300200 Timisoara, Romania; larisaciudin0389@gmail.com; 4 Doctoral School of Biological and Biomedical Sciences, University of Oradea, 410087 Oradea, Romania; 5 Medical Informatics and Biostatistics, Department of Functional Sciences, Faculty of Medicine, “Victor Babes” University of Medicine and Pharmacy, 300041 Timisoara, Romania; 6 Physiology, Department of Functional Sciences, Faculty of Medicine, “Victor Babes” University of Medicine and Pharmacy, 300041 Timisoara, Romania; patrascu.raul@umft.ro; 7 General Medicine, Faculty of Medicine, “Victor Babes” University of Medicine and Pharmacy, 300041 Timisoara, Romania; paula.roman@student.umft.ro; 8 Genomic Medicine Centre, “Victor Babes” University of Medicine and Pharmacy, 300041 Timisoara, Romania

**Keywords:** reproductive genetic carrier screening, autosomal recessive disorders, pathogenic variants, couple-based screening, bioethics

## Abstract

Reproductive genetic carrier screening (RGCS) is recommended preconceptionally or early pregnancy to identify the risk of autosomal recessive (AR) disorders in healthy couples. Data on shared carrier status at the couple’s level remains limited in Eastern Europe. This study presents the first couple-based RGCS analysis in Western Romania. We retrospectively analyzed RGCS results from 247 couples with no known consanguinity at the time of evaluation (494 apparently unrelated individuals, aged 22–52 years), assessed at a single genetic center between 2020–2024. Next-generation sequencing was performed using an expanded panel targeting 302 genes, including 300 genes associated with AR onset disorders. This analysis was accompanied by both pre- and post-test genetic counseling. The prevalence of individual and shared carrier status and reproductive risk was assessed. Pathogenic or likely pathogenic (PLP) variants were identified in the study cohort, with an overall couple carrier frequency of 64.37%. Shared carrier status for PLP variants in the same gene was identified in 17.4%, including three couples carrying pathogenic variants in two shared genes. Additionally, 46.96% of couples carried pathogenic variants in different genes without overlapping. The most frequently shared genes with PLP variants were *HFE*, *CFTR*, *SMN1*, *BTD*, and *COL7A1*; 14 additional shared genes with PLP variants were associated with severe, early-onset disorders. Forty-three couples were deemed high risk for AR conditions. Their reproductive choices varied, including in vitro fertilization or proceeding with pregnancy with or without prenatal testing. Couple-based RGCS revealed a substantial burden of shared AR carrier status in Western Romania, supporting the implementation of population-level screening programs to improve reproductive risk assessment and informed decision-making.

## 1. Introduction

EURORDIS estimates that 6–8% of the population are affected by a rare disease [1], with autosomal recessive (AR) disorders accounting for a substantial proportion of this burden. Although individually rare, collectively these conditions represent a significant public health concern due to their cumulative prevalence and impact on morbidity and mortality. About 2–4% of couples are at risk of conceiving a child with an autosomal recessive genetic disorder [2]. RGCS has emerged as a key strategy for identifying couples at risk of having affected offspring and for supporting informed reproductive decision-making.

Professional societies have progressively expanded their recommendations regarding carrier screening. In 2017, the American College of Obstetricians and Gynecologists (ACOG) issued a committee opinion supporting expanded carrier screening panels, recommending testing for conditions with a carrier frequency above 1%, which, in the referenced study, corresponded to variants in 40 genes and enabled the identification of 76–97% of carrier couples [3]. Historically, carrier screening initiatives began in the 1970s within the Ashkenazi Jewish population, primarily targeting Tay–Sachs disease and enabling substantial reductions in disease incidence through informed reproductive choices [4].

Subsequently, carrier screening focused on specific disorders known to have a higher prevalence in certain ethnic populations [5]. Cyprus historically established one of the first national carrier screening programs for beta-thalassemia, with premarital screening reducing disease incidence dramatically [6]. The carrier screening program in Turkey is available as the Premarital Screening Program for Hemoglobinopathy and Spinal muscular atrophy (SMA). Encouragingly, in regions with a high prevalence of certain AR conditions (for example, beta-thalassemia and sickle cell disease), single-gene screening programs have demonstrated remarkable reductions in the frequency of affected births [7].

Currently, the scope of carrier screening varies considerably between countries, ranging from a limited number of conditions to extensive panels. However, in many settings, carrier screening is not publicly funded and access remains unequal [3]. The implementation of population-wide screening programs based on publicly funded whole-exome sequencing (WES) technologies has been proposed as a means to ensure equitable access, yet such initiatives remain limited [8].

In Romania, reproductive genetic carrier screening is not part of a national public health program, and available testing is largely opportunistic and privately funded. For couples who do not belong to high-risk ethnic groups, do not originate from genetically isolated regions, and have no known family history of AR disorders, expanded panel screening may represent a more appropriate strategy than ethnicity-based approaches. Nevertheless, data regarding the feasibility, detection rate, and clinical utility of couple-based carrier screening in the Romanian population are lacking.

Therefore, the primary aim of this study was to identify PLP variants and, specifically, to detect couples in which both partners are heterozygous carriers of PLP variants in the same gene. Through this identification process, the reproductive risk could be inferred, enabling the provision of targeted genetic counseling, including discussion of appropriate reproductive options.

## 2. Results

### 2.1. Indications for Genetic Screening

A total of 247 couples were included in the study. At the time of testing, 37.65% of the female partners were pregnant. All couples underwent an expanded carrier screening panel comprising 302 genes, including 300 genes associated with AR conditions and two genes (F2 and F5) linked to autosomal dominant (AD) disorders, which were not included in the present analysis. In all couples, both partners underwent carrier screening.

A history of reproductive complications represented another major indication for testing. Specifically, 37.25% of couples reported between one and four previous miscarriages. In addition, 10.12% of couples had a history of unsuccessful in vitro fertilization (IVF), with the number of failed procedures ranging from 1 to 15.

Genetic evaluation was also indicated for couples with a known family history of genetic disorders. Overall, 9.71% of couples were referred due to a previously affected child or a relative diagnosed with an autosomal recessive PLP-related disorder.

Among them, 21 couples had children affected by genetic diseases; in 19 families, the diagnosis corresponded to an autosomal recessive monogenic disorder. Six of these affected children were deceased at the time of parental testing. In this cohort, mortality was associated with pathogenic variants in genes including *SMN1*, *CFTR*, and *COL7A1*, as well as seven other genes (Table 1).

Additionally, 5.27% of couples underwent preconception carrier screening despite the absence of previous reproductive complications or a known family history of genetic disease.

### 2.2. Classification of Couples According to the Presence of PLP Variants

**Figure 1:**
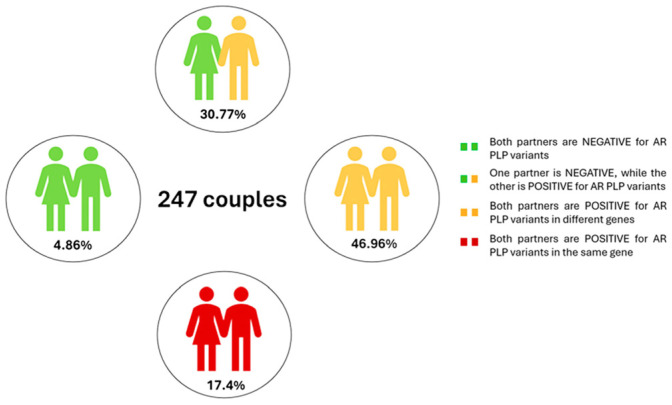
Couples categorized according to reproductive genetic risk, with color coding reflecting the presence of pathogenic or likely pathogenic (PLP) AR variants in each partner of the couple.

We classified couples into two main categories, Negative and Positive, based on the presence of PLP variants in their genotypic profile. Couples in which both partners were negative were identified in 4.86% of the cases (Figure 1).

A total of 95.14% of couples had at least one partner carrying a PLP variant. These couples were further classified as follows: 76 couples had only one partner identified as a carrier, 116 couples had both partners carrying PLP variants in different genes, and 43 couples had both partners carrying PLP variants in the same gene. The remaining 12 couples (4.86%) were negative in both partners.

### 2.3. Classification of Genes According to Reproductive Risk

**Figure 2:**
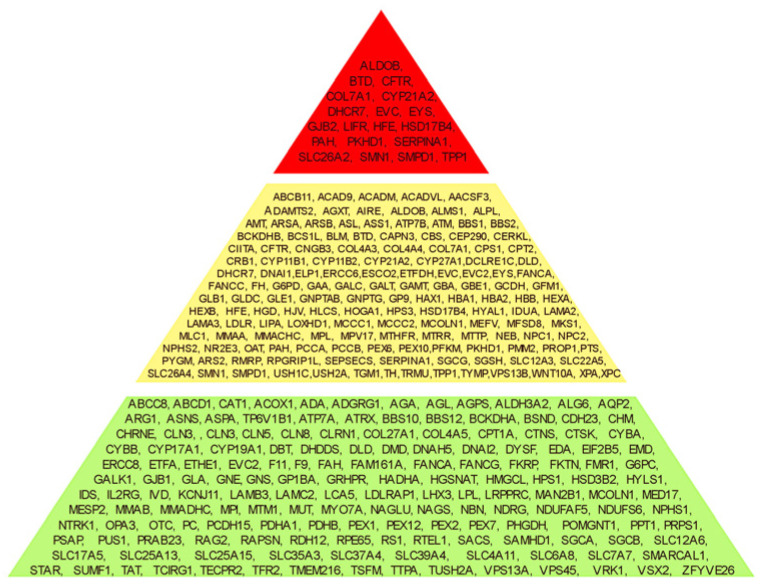
Gene classification according to the reproductive risk.

Genes were classified according to the presence of PLP variants in couples in order to assess reproductive risk for autosomal recessive (AR) disorders. A total of 19 genes were identified in which PLP variants were present in both partners of 43 couples, placing them at increased reproductive risk (red zone). In contrast, 142 genes harbored PLP variants in only one partner across 192 couples, with no shared variants, indicating no reproductive risk (yellow zone). For the remaining 157 genes, no PLP variants were detected in any individual, suggesting that these disorders pose minimal risk in the studied population (green zone) (Figure 2).

The highest proportion of reproductive risk was attributable to three genes, namely *HFE*, *CFTR*, and *SMN1*, which together accounted for 65.12% of all at-risk couples, each case involving both partners carrying heterozygous PLP variants in the same gene. When analyzed individually, *HFE* was identified in 32.56% of cases, *CFTR* in 18.60%, and *SMN1* in 13.95%, with all couples harboring concordant heterozygous PLP variants in the respective gene.

The intermediate-frequency category included *BTD* and *COL7A1*, each responsible for 4.65% of at-risk couples, all cases being explained by the presence of heterozygous PLP variants in both partners within the same gene.

**Figure 3:**
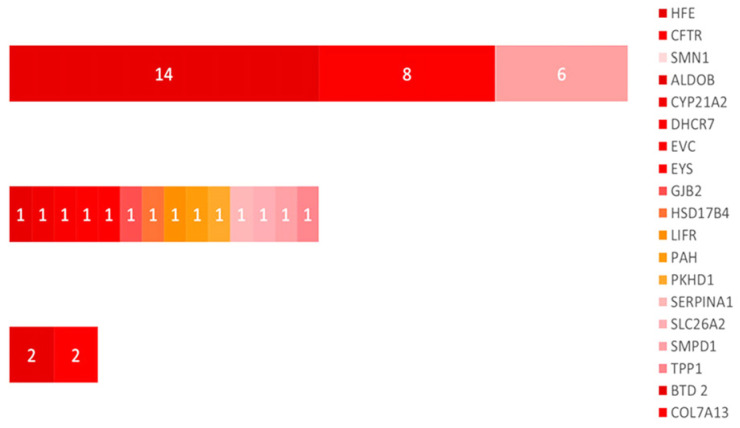
Risk categories according to the number of couples identified with shared genes.

The remaining genes, namely *ALDOB*, *CYP21A2*, *DHCR7*, *EVC*, *EYS*, *GJB2*, *HSD17B4*, *LIFR*, *PAH*, *PKHD1*, *SERPINA1*, *SLC26A2*, *SMPD1*, and *TPP1*, were each identified in 2.33% of at-risk couples, with reproductive risk determined by the detection of heterozygous PLP variants in both members of the couple in the corresponding gene. Collectively, this genetically heterogeneous group accounted for 32.56% of the total at-risk cohort (Figure 3).

### 2.4. Couples with PLP Variants in Shared Genes

A total of 43 couples carried pathogenic or likely pathogenic (PLP) variants in the same gene, indicating an increased reproductive risk for autosomal recessive disorders Most couples had variants in a single shared gene, while a few (couples 16, 23, and 27) presented with variants in two genes. Both partners carried identical variants in several cases (e.g., CFTR, GJB2, BTD, SMN1), whereas in others, different variants were identified within the same gene, consistent with potential compound heterozygosity. Recurrent findings included variants in HFE, CFTR, and SMN1, reflecting their known carrier frequency (Table 2).

For SMPD1 (couple 1), the notation c.[variant];[?] indicates that a variant was identified on one allele, while the second allele could not be characterized.

### 2.5. Gene Classification According to Disease Morbidity

We analyzed the at-risk couples by classifying the genes carrying PLP variants according to the morbidity of the associated genetic conditions, which were grouped into high, moderate, and low morbidity categories.

The first category of high morbidity diseases comprised nine genes, corresponding to 15 at-risk couples (Table 3). It should be noted that for six genes listed (*PKHD1*, *SMN1*, *COL7A1*, *TPP1*, *HSD17B4*, *SMPD1*), couples sought genetic testing because they already had an affected child, totaling 12 affected families (Table 1). These genes are associated with disorders characterized by severe clinical manifestations and high risk of morbidity and mortality.

The second category of moderate-morbidity diseases included six genes, corresponding to 11 at-risk couples. An affected child had already been born in six families due to variants in three genes: *CFTR* (4 cases), *PAH* (1 case), and *ALDOB* (1 case) (Table 3). These genes are associated with conditions of intermediate severity, with moderate morbidity that may require ongoing clinical management.

The third category of low-morbidity diseases comprised four genes, corresponding to 18 at-risk couples. Except for the *LIFR* gene, which was identified in one family with an affected child, the remaining variants in this category were detected incidentally through carrier screening in both partners, representing unexpected findings. These genes are associated with conditions that generally result in mild clinical manifestations and low morbidity (Table 3).

### 2.6. Medically Actionable Conditions

We classified genes according to their association with AR conditions for which interventions are available that may prevent disease occurrence or reduce disease severity through treatment, surveillance, or reproductive options (Table 4).

The clearly medically actionable category includes genes associated with disorders for which effective treatment, prevention, or disease management strategies are available. We identified 10 genes associated with conditions of high morbidity, for which interventions can significantly reduce clinical impact and improve patient outcomes.

The partially actionable or context-dependent category comprises for 4 genes for which interventions exist but are limited, supportive, or mutation-specific. Conditions associated with these genes generally result in moderate morbidity, requiring ongoing management or mutation-specific approaches rather than fully preventive or curative options.

The currently limited clinical actionability category includes genes for which no established therapy exists to modify disease progression or provide prevention. Management is primarily symptomatic or supportive, and gene-specific therapies remain experimental or unavailable. These 5 genes are generally associated with low morbidity conditions, although they may still have significant implications for affected families.

### 2.7. Regional Origins of the Study Cohort Across Romania

The study cohort comprised 494 individuals, who, at the time of evaluation, resided or worked in the western region of Romania, primarily in Timis County. Analysis of their self-reported geographical origin revealed a diverse representation from multiple counties (Table 5). Most participants (77.53%) reported originating from Timis County, while the remaining individuals were distributed across 20 other Romanian counties, with the largest contributions from Arad (5.47%), Caras-Severin (3.85%), and Hunedoara (2.63%). Additional counties contributed smaller proportions (≤1.82% each), and four participants (0.81%) reported a foreign origin (Table 5).

## 3. Discussion

Population-level carrier screening supported by governmental resources has not yet been implemented in Romania. To our knowledge, this study represents the first couple-based RGCS analysis performed in a single-center from a private practice in Western Romania and provides an overview of the potential implications of these findings. Given that the real prevalence of AR conditions remains largely unknown across specific geographical regions [28], our results contribute updated data for the western region of Romania.

The highest proportion of reproductive risk was attributable to three genes, namely *HFE*, *CFTR*, and *SMN1*, in which PLP variants were identified, accounting together for 65.12% of all at-risk couples. When analyzed individually, PLP variants in *HFE* were identified in 32.56% of cases, *CFTR* in 18.60%, and *SMN1* in 13.95%. In all these situations, both partners were heterozygous carriers of PLP variants in the same gene. The intermediate-frequency category included PLP variants in *BTD* and *COL7A1*, each responsible for 4.65% of at-risk couples. The remaining 14 genes, namely *ALDOB*, *CYP21A2*, *DHCR7*, *EVC*, *EYS*, *GJB2*, *HSD17B4*, *LIFR*, *PAH*, *PKHD1*, *SERPINA1*, *SLC26A2*, *SMPD1*, and *TPP1*, harbored PLP variants that were each identified in 2.33% of at-risk couples. Collectively, this genetically heterogeneous group accounted for 32.56% of the total at-risk cohort, as illustrated in Figure 3.

A previous study of the western population in Romania had identified the risk associated with the most frequent genes and related autosomal recessive (AR) monogenic disorders [29]. Our current study, targeting non-consanguineous couples, highlights unexpected differences in reproductive risk. The most frequent PLP variants in our cohort were found in *HFE* (1:5), *CFTR* (1:9), *BTD* (1:16), *GJB2* (1:17), and *CYP21A2* (1:19). However, regarding RGCS, the most shared genes within couples were *HFE*, *CFTR*, and *SMN1*, cumulatively accounting for 65.12% of at-risk couples, followed by *BTD* and *COL7A1* (9.3%). These results indicate that population-level carrier frequency provides an overview of potential risk, but accurate reproductive risk assessment requires identification of shared variants within specific couples.

Genes such as *SMN1* and *COL7A1* are associated with conditions characterized by high morbidity and mortality (Table 1 and Table 2). Although their carrier frequencies were not among the highest, eight affected families were identified, highlighting the considerable clinical impact of these disorders. Overall, the AR conditions already diagnosed in our cohort were linked to *SMN1*, *COL7A1*, *TPP1*, *HSD17B4*, *SMPD1*, *LIFR*, and *PKHD1*, underscoring the substantial burden these diseases impose on affected families and communities [30].

The discussion expands, arguing that the composition of genetic screening panels should not be limited to genes associated with high morbidity, nor should it be determined exclusively by clinicians. Instead, it should incorporate the perspectives of a broad range of relevant groups, including individuals who would use such testing (prospective parents) as well as patients with lived experience of the conditions under consideration [31].

Although PLP variants were identified in most individuals, fewer than 20% of couples were truly at reproductive risk. Among these at-risk couples, 42.85% had previously affected children (Table 1). These families sought genetic testing primarily to inform future reproductive decisions, as raising a child with a hereditary disorder can impose considerable emotional, social, and financial burdens [32].

Previous studies using smaller gene panels estimated that approximately 1–2% of reproductive couples are at increased risk for AR disorders [33]. In our cohort, the 300-gene panel identified 235 positive carrier couples (95.14%), while only 12 couples (4.85%) tested negative in both partners. Among couples carrying at least one PLP variant, 158 (64.23%) were identified as carriers, including 43 couples (17.4%) in which both partners carried PLP variants in the same gene. These findings closely resemble those reported in a study from Portland, USA, using a 728-gene panel, where approximately 17% of couples shared at least one carrier gene [5]. An Italian study conducted on 766 couples from an IVF clinic identified 173 couples (22.6%) in which one partner was a carrier, whereas 20 couples (2.6%) were found to be at increased reproductive risk, carrying pathogenic or likely pathogenic variants in the *CFTR*, *FMR1* (*FRAXA*), *SMN1*, *HBB*, and *DHCR7* genes [34].

Identical variants were detected in 60.46% of at-risk couples, whereas 39.53% carried different variants within the same gene (Figure 2). Even in non-consanguineous couples, such situations may result in unexpected reproductive risk. Some variants, including those in *HFE*, *CFTR*, *SMN1*, *BTD*, and *GJB2*, are relatively common in European populations due to shared ancestry [29]. In contrast, the rare variants identified in *COL7A1*, *ALDOB*, *EVC*, *EYS*, and *HSD17B4* may reflect distant familial relationships or unknown and certainly unexpected founder effects, highlighting the importance of detailed pedigree analysis.

The exact list of identical variants, as detected in both the mother and the father, can be consulted in Table 2. The reproductive risk in non-consanguineous couples, although generally low [8], may be unpredictable. Increased reproductive risk was observed in several couples originating from geographically proximate areas who had previously delivered affected children. In all cases in which carrier status is identified, discussion with relatives and targeted genetic counseling are recommended in order to guide future reproductive planning.

When comparing the PLP variants identified in our cohort with those reported in a previous study on the Western Romanian population, we observed that the high-frequency gene group (<1:50 carriers) included PLP variants in *HFE*, *CFTR*, *BTD*, *GJB2*, *CYP21A2*, *SERPINA1*, *PAH*, and *SMN1*; the moderate-frequency group (1:51–1:100) included PLP variants in *ALDOB*, *COL7A1*, *DHCR7*, *EVC*, *SLC26A2*, and *TPP1*; the low-frequency group (1:101–1:150) included PLP variants in *EYS*; whereas the extremely rare gene group (>1:151) included PLP variants in *HSD17B4*, *LIFR*, *PKHD1*, and *TPP1* [29]. Notably, rare conditions caused by genes belonging to the so-called “long tail,” often associated with high morbidity, still affected approximately 2% of the tested couples, emphasizing the importance of comprehensive RGCS.

High-morbidity genes identified in this cohort (PKHD1, SMN1, COL7A1, TPP1, HSD17B4, CYP21A2, DHCR7, SMPD1, and EYS) can be broadly categorized according to clinical actionability (Table 3). Some conditions are considered medically actionable when treatment is initiated immediately after birth (such as SMN1, CYP21A2, TPP1), others are partially actionable (PKHD1, HSD17B4, SMPD1, COL7A1), whereas several genes currently fall under the category of limited clinical actionability (DHCR7, EYS) (Table 4).

Couples in which only one partner is a carrier generally do not face an increased risk of having an affected child, although genetic counseling is still recommended. When both partners carry variants in the same gene, the risk of an affected child is typically 25% per pregnancy, with a 50% probability of carrier offspring and a 25% probability of unaffected offspring. In such cases, couples should be informed about the available reproductive options to enable informed decision-making. Possible reproductive strategies include:

(1) Natural conception followed by prenatal diagnosis, typically through amniocentesis. If the fetus is affected, parents may prepare for the birth of a child with the condition, or consider pregnancy termination, depending on legal regulations and personal beliefs.
(2) IVF with preimplantation genetic testing for monogenic disorders (PGT-M) prior to embryo transfer, allowing the selection and implantation of unaffected embryos. Although this approach prevents the establishment of an affected pregnancy, it involves substantial costs, maternal hormonal treatment, and potential ethical considerations.
(3) Use of donor gametes (sperm or oocytes) from donors genetically screened for carrier status, thereby eliminating the risk for the specific condition.
(4) Adoption as a non-biological parenting option.
(5) Choosing not to pursue biological parenthood, an option selected by some couples.

After receiving the RGCS results, reproductive decisions may vary considerably, being influenced by the highly personal context of each couple, the presence of relatives affected by the respective condition, social norms regarding disability, the availability of treatment options, and the perspectives of clinicians managing individuals with the condition [35]. During post-test genetic counseling, patients were informed about diseases associated with the identified PLP variants and about available reproductive options; some couples who later pursued another pregnancy opted for prenatal diagnosis through amniocentesis followed by targeted genetic testing. Personalized genetic counseling should consider reproductive history, family background, existing genetic data, and the couple’s willingness to undergo preconceptional or prenatal testing [36]. In our cohort, a high proportion of women were already pregnant at the time of testing, highlighting the importance of pre-test counseling in providing accurate information and managing anxiety related to genetic testing [37]. Ideally, RGCS should be offered preconceptionally or early in pregnancy [38].

Reproductive-age couples who receive a result indicating an increased risk of having a child affected by a severe childhood-onset genetic disorder, are unlikely to have prior knowledge of, or experience with, that condition. For this reason, post-test counseling should be readily accessible and should provide clear information about the disorder and its expected clinical manifestations, so that prospective parents can make decisions aligned with their values and expectations. Face-to-face counseling is considerably more useful, and in many cases indispensable, compared with information available online, which often cannot convey a realistic understanding of what it means to raise a child with the condition or what life may be like for the affected individual [36,39].

In Romania, access to RGCS remains limited, as these tests are currently available mainly through private laboratories and are not reimbursed by the national health system. As in many other countries, limited awareness and financial constraints represent important barriers to wider implementation [37,40].

Recent public health initiatives suggest an increasing national interest in expanding genetic screening programs. In February 2026, during Rare Disease Day, the Romanian Minister of Health announced the expansion of the national neonatal screening program from three to twenty-two conditions and the establishment of a National Neonatal Screening Registry. The program introduces 19 additional metabolic and genetic biomarkers that allow early detection of severe and rare diseases before irreversible damage occurs. This measure represents an important step toward strengthening preventive healthcare policies and aligning national practices with European standards [41]. Within this context, our cohort, although consisting of participants established and tested in Western Romania, reflects geographic origins that extend across the country, providing initial insights into the genetic background of the Romanian population.

These developments highlight the growing recognition of the importance of early genetic diagnosis in Romania and support the need for broader implementation of carrier screening strategies, including RGCS, as part of comprehensive reproductive and preventive healthcare programs.

The 300 autosomal-recessive–associated genes can be conceptualized as a pyramid: 157 genes were not identified in any individual (forming the base), 143 genes were detected in at least one partner (middle level), and 19 genes were associated with reproductive risk (the apex of the pyramid) (Figure 2). Ensuring the inclusion of these 19 genes in screening panels may be particularly important for effective population screening and for the development of future public health strategies.

Globally, government-funded carrier screening programs vary substantially in scope and implementation. Australia has introduced publicly funded screening for three conditions starting in 2024 [38], while Cyprus and Greece have long-standing national prevention programs targeting beta-thalassemia. The United Kingdom offers universal antenatal screening for beta-thalassemia [42], and Israel has recently expanded its national carrier screening program to include 290 genes and approximately 650 pathogenic variants [3]. In parallel, pilot studies are currently being conducted worldwide to address the practical, psychosocial, and bioethical challenges associated with the implementation of RGCS programs [43]. As genomic technologies become increasingly accessible and public awareness continues to grow, RGCS is expected to become a routine component of reproductive planning, regardless of an individual’s genetic background, ethnicity, family history, or the availability of a government-funded screening program [39].

Based on the current level of evidence, and considering factors such as healthcare provider time, overall healthcare costs, the frequency of severely affected offspring, patient satisfaction, and, most importantly, the diagnostic yield in identifying at-risk couples or pregnancies, several authors recommend that RGCS should be offered to all couples who are planning a pregnancy or who already have an ongoing pregnancy [34,44]. Expanding access to comprehensive carrier screening may therefore represent an important step toward improving reproductive autonomy, enabling informed decision-making, and supporting future public health strategies aimed at reducing the burden of severe inherited disorders.

## 4. Materials and Methods

### 4.1. Participant Selection and Clinical Data

A retrospective study was conducted on data obtained from 494 apparently unrelated individuals (247 couples) of reproductive age (22–52 years). All participants were tested between 1 January 2020 and 30 December 2024 at a single private genetic center from Western Romania.

All couples underwent expanded carrier screening using a multigene panel that included 302 genes, of which 300 were associated with autosomal recessive inheritance. The present analysis included only PLP variants identified in autosomal recessive genes.

Carrier screening was performed for both partners in all couples.

The following clinical and demographic data were recorded for each participant: age at testing, county of origin, reproductive history, relevant family history of genetic disorders, and the gene and variant identified in each individual.

### 4.2. Genetic Counseling

All participants underwent two sessions of genetic counseling conducted by an experienced clinical geneticist. During the pre-test session, reproductive history, parental age, and any known or suspected personal or family hereditary conditions were documented, and detailed family pedigrees were constructed. After the carrier screening results became available, a post-test counseling session was held to communicate the identified reproductive risks and their potential clinical consequences. For couples classified as high risk, possible reproductive options were discussed, with the main recommendation being prenatal genetic diagnosis by amniocentesis in future pregnancies, followed by targeted confirmation of the pathogenic variants identified in the parents.

### 4.3. Carrier Screening and Gene Panel

Carrier screening was performed using the Invitae Comprehensive Carrier Screen (Invitae Corporation, San Francisco, CA, USA), comprising the analysis of 302 genes, including 300 associated with autosomal recessive conditions and two genes (*F2* and *F5*) associated with autosomal dominant conditions. The complete gene list is available in Figure S1 (Supplementary Materials).

Peripheral blood samples were collected in EDTA tubes (BD Vacutainer®, Becton, Dickinson and Company, Franklin Lakes, NJ, USA) from all individuals at the Medical Genetics Office Doctor Gug (Timișoara, Romania) and shipped to Invitae Corporation (San Francisco, CA, USA), where all genetic analyses were performed under a contractual agreement.

Genomic DNA extraction, library preparation, and target enrichment were performed by Invitae Corporation using a hybridization-based capture protocol. Next-generation sequencing (NGS) was carried out on Illumina sequencing platforms (Illumina, Inc., San Diego, CA, USA), according to the laboratory’s clinically validated protocols.

Sequencing reads were aligned to the human reference genome (GRCh37) using a proprietary bioinformatics pipeline developed and validated by Invitae Corporation. Variant calling, annotation, and interpretation were performed using the same validated internal pipeline, in accordance with the standards implemented in the laboratory at the time of testing.

As detailed information regarding specific software tools, versions, and algorithms is proprietary, these data are provided and validated by Invitae Corporation (San Francisco, CA, USA). For further technical details, the laboratory can be contacted at clientservices@invitae.com.

The analysis targeted coding regions, ±10 base pairs of flanking intronic sequences, and selected clinically relevant non-coding regions included at the time of panel design [45].

Copy number variants (CNVs) were detected using read-depth–based algorithms implemented within the validated pipeline of Invitae Corporation. Genes with complex genomic architecture were further analyzed using gene-specific approaches, including long-range PCR and/or long-read sequencing when required. Repeat expansions were assessed using PCR-based methods followed by capillary electrophoresis, as specified by the testing laboratory [45].

Only clinically significant variants (pathogenic or likely pathogenic) were included in the analysis, while variants of uncertain significance (VUS) were not reported unless subsequently reclassified. Variant classification and interpretation were conducted according to the joint guidelines of the American College of Medical Genetics and Genomics and the Association for Molecular Pathology (ACMG/AMP), as previously described [45].

A negative result does not exclude carrier status; therefore, residual risk was estimated by Invitae Corporation based on detection rates and population-specific carrier frequencies, as specified in the laboratory report.

### 4.4. Assay Limitations

Based on validation data provided by Invitae Corporation (San Francisco, CA, USA), the assay demonstrates >99% analytical sensitivity and specificity for single nucleotide variants and small insertions/deletions (<15 bp), as well as exon-level copy number variants.

Larger insertions/deletions and certain copy number changes are also detectable; however, sensitivity may be reduced depending on variant size, genomic context, and sequence complexity. Copy number analysis is performed at single-exon resolution, although rare events affecting individual exons may not be reliably detected due to sequence-specific or technical limitations [45].

Certain classes of variants, including structural rearrangements (e.g., inversions, translocations, or gene conversion events), as well as variants located in regions with complex genomic architecture (e.g., segmental duplications or short tandem repeats), may not be detected. In addition, limitations inherent to short-read sequencing technologies (Illumina platforms, Illumina, Inc., San Diego, CA, USA) may prevent accurate determination of variant phasing, low-level mosaicism, or mapping in highly homologous regions.

Unless specifically targeted, promoter regions and other non-coding sequences are not systematically analyzed. Furthermore, the use of the GRCh37 reference genome instead of newer assemblies (e.g., GRCh38) may limit the representation of certain genomic regions.

This assay analyzes genomic DNA extracted from peripheral blood; in rare situations, the analyzed DNA may not fully reflect the individual’s constitutional genome (e.g., in cases of chimerism, prior transplantation, or recent transfusion) [45].

All performance metrics and technical specifications are based on internal validation studies conducted by Invitae Corporation and reported in the official laboratory documentation.

### 4.5. Statistical Analysis and Cohort Characteristics

The study cohort comprised 494 individuals, representing 247 couples who underwent RGCS upon request, outside of a national screening program. The age of participants ranged between 22 and 52 years, with a mean age of 33.93 ± 5.48 years and a median of 34 years, reflecting a population within the typical reproductive age range.

Reproductive history data were recorded for descriptive purposes. A proportion of couples reported previous pregnancy losses (range: 0–4), while a smaller subset had a history of unsuccessful IVF attempts (range: 0–15 procedures).

These variables were collected to provide clinical context regarding the study population but were not used for inferential statistical analysis, as they are not directly related to the genetic outcomes assessed in this study.

## 5. Conclusions

This study represents the first couple-based RGCS analysis in Western Romania, highlighting its utility in identifying reproductive risk even among non-consanguineous couples without a known family history of monogenic disorders. Among 247 couples, 17.4% carried shared PLP variants in the same gene, with *HFE*, *CFTR*, and *SMN1* accounting for the majority of at-risk couples, while rare high-morbidity variants in genes such as *COL7A1*, *ALDOB*, and *TPP1* also contributed to reproductive risk. These findings underscore the importance of assessing couple-level shared variants, not just individual carrier status, for accurate reproductive risk evaluation.

RGCS enables informed reproductive decision-making by providing prospective parents with actionable information regarding autosomal recessive condition. Comprehensive pre- and post-test counseling is essential to explain potential clinical outcomes, available interventions, and reproductive options, even extremely rare variants, often overlooked in smaller panels, affected approximately 2% of couples, emphasizing the value of comprehensive gene panels.

At the population level, these results support integrating RGCS into routine reproductive care in Romania, where national programs are currently limited. As genomic technologies become more accessible and public awareness increases, RGCS is expected to become a standard component of reproductive planning. Broad implementation could enhance reproductive autonomy, reduce the incidence of severe autosomal recessive disorders, and inform future public health strategies.

In conclusion, this study demonstrates that even in a relatively small and genetically heterogeneous population, RGCS effectively identifies at-risk couples, providing actionable data to guide reproductive decisions and laying the groundwork for national-level screening initiatives and further research in Eastern Europe.

## Figures and Tables

**Table 1:** Families with an affected child at the time of carrier testing.

Gene	Disease	Number of Affected Families	Number of Deceased Children
*SMN1*	Spinal muscular atrophy (SMA)	6	1
*CFTR*	Cystic fibrosis (CF)	4	0
*COL7A1*	Dystrophic epidermolysis bullosa	2	1
*TPP1*	Neuronal ceroid lipofuscinosis type 2	1	1
*HSD17B4*	Peroxisomal metabolic conditions	1	1
*SMPD1*	Niemann–Pick disease type A	1	1
*LIFR*	Stüve–Wiedemann syndrome	1	1
*PKHD1*	AR polycystic kidney disease	1	1
*PAH*	Phenylketonuria	1	0
*ALDOB*	Hereditary fructose intolerance	1	0

**Table 2:** Common genes and PLP gene variants identified in at-risk couples.

Couple No.	Shared Genes	Gene PLP Variant
1	*SMPD1*	c.[1268A>G];[?] (p.[His423Arg];[?])
	*SMPD1*	c.[1685T>A];[?] (p.[Met562Lys];[?])
2	*SMN1*	Heterozygous Deletion of Exons 7 + 8
	*SMN1*	Heterozygous Deletion of Exons 7 + 8
3	*LIFR*	c.1789C>T (p.Arg597*^1^)
	*LIFR*	c.1418del (p.Ser473Leufs*15)
4	*HFE*	c.845G>A (p.Cys282Tyr) § ^2^
	*HFE*	c.187C>G (p.His63Asp) §
5	*CFTR*	c.1210-34TG[11]T[5] §
	*CFTR*	c.1210-34TG[11]T[5] §
6	*HFE*	c.187C>G (p.His63Asp) §
	*HFE*	c.187C>G (p.His63Asp) §
7	*SLC26A2*	c.1957T>A (p.Cys653Ser)
	*SLC26A2*	c.1724del (p.Lys575Serfs*10)
8	*HFE*	c.187C>G (p.His63Asp) §
	*HFE*	c.187C>G (p.His63Asp) §
9	*HFE*	c.187C>G (p.His63Asp) §
	*HFE*	c.187C>G (p.His63Asp) §
10	*HFE*	c.845G>A (p.Cys282Tyr) §
	*HFE*	c.187C>G (p.His63Asp) §
11	*GJB2*	c.35del (p.Gly12Valfs*2)
	*GJB2*	c.35del (p.Gly12Valfs*2)
12	*EYS*	c.9036del (p.Leu3013Serfs*6)
	*EYS*	c.9036del (p.Leu3013Serfs*6)
13	*HFE*	c.187C>G (p.His63Asp) §
	*HFE*	c.845G>A (p.Cys282Tyr) §
14	*EVC*	c.919T>C (p.Ser307Pro)
	*EVC*	c.919T>C (p.Ser307Pro)
15	*HFE*	c.187C>G (p.His63Asp) §
	*HFE*	c.187C>G (p.His63Asp) § homozygous
16	*CFTR* and *HFE*	c.1210-34TG[11]T[5] (Intronic) § and c.187C>G (p.His63Asp) §
	*CFTR* and *HFE*	c.1210-34TG[11]T[5] (Intronic) § and c.187C>G (p.His63Asp) §
17	*HFE*	c.187C>G (p.His63Asp) §
	*HFE*	c.187C>G (p.His63Asp) §
18	*DHCR7*	c.91C>T (p.Arg31Cys)
	*DHCR7*	c.452G>A (p.Trp151*)
19	*BTD*	c.1330G>C (p.Asp444His)
	*BTD*	c.1330G>C (p.Asp444His)
20	*CFTR*	c.3909C>G (p.Asn1303Lys)
	*CFTR*	c.1521_1523del (p.508del)
21	*HFE*	c.187C>G (p.His63Asp) §
	*HFE*	c.187C>G (p.His63Asp) §
22	*SMN1*	Heterozygous Deletion of Exons 7 + 8
	*SMN1*	Heterozygous Deletion of Exons 7 + 8
23	*CYP21A2* and *HFE*	c.955C>T (p.Gln319*) and c.187C>G (p.His63Asp) §
	*CYP21A2* and *HFE*	c.844G>T (p.Val282Leu) and c.187C>G (p.His63Asp) §
24	*HFE*	c.187C>G (p.His63Asp) §
	*HFE*	c.187C>G (p.His63Asp) §
25	*HFE*	c.187C>G (p.His63Asp) §
	*HFE*	c.187C>G (p.His63Asp) §
26	*HFE*	c.187C>G (p.His63Asp) §
	*HFE*	c.845G>A (p.Cys282Tyr) §
27	*BTD* and *SERPINA1*	c.1330G>C (p.Asp444His) and c.863A>T (p.Glu288Val) §
	*BTD* and *SERPINA1*	c.1595C>T (p.Thr532Met) and c.1177C>T (p.Pro393Ser)
28	*SMN1*	Heterozygous Deletion of Exons 7 + 8
	*SMN1*	Heterozygous Deletion of Exons 7 + 8
29	*CFTR*	c.1521_1523del (p.508del)
	*CFTR*	c.1521_1523del (p.508del)
30	*SMN1*	Heterozygous Deletion of Exons 7 + 8 in SMN1 and Duplication of SMN2
	*SMN1*	Heterozygous Deletion of Exons 7 + 8 in SMN1 and Duplication of SMN2
31	*HSD17B4*	c.788del p.(Pro263Glnfs*2)
	*HSD17B4*	c.788del p.(Pro263Glnfs*2)
32	*ALDOB*	c.448G>C (p.Ala150Pro)
	*ALDOB*	c.448G>C (p.Ala150Pro)
33	*CFTR*	c.1521_1523del (p.508del)
	*CFTR*	c.1521_1523del (p.508del)
34	*TPP1*	c.622C>T, p.(Arg208*)
	*TPP1*	c.1678_1679delCT
35	*CFTR*	c.1521_1523del (p.508del)
	*CFTR*	c.3472 C>G (Arg1158*)
36	*PAH*	c.1066-11G>A
	*PAH*	c.1222C>T p.Arg408Trp
37	*CFTR*	c.1521_1523del (p.508del)
	*CFTR*	7T + 7T
38	*CFTR*	c.1521_1523del (p.508del)
	*CFTR*	c.3909C>G (p.Asn1303Lys)
39	*SMN1*	Heterozygous Deletion of Exons 7 + 8
	*SMN1*	Heterozygous Deletion of Exons 7 + 8
40	*SMN1*	Heterozygous Deletion of Exon 7
	*SMN1*	Heterozygous Deletion of Exons 7 + 8
41	*COL7A1*	c.425A>G (p.Lys142Arg) (Exon 3)
	*COL7A1*	c.2308_c.2314 + 1delAGGACTGG (Intron 17)
42	*COL7A1*	c.3140-2A>G
	*COL7A1*	c.3140-2A>G
43	*PKHD1*	Exon 3 (c.107C>T) p.Thr36Met
	*PKHD1*	Exon 64 (c.11439C>G) p.Phe3813Leu

*^1^ Premature stop codon; § ^2^ This variant is known to have low penetrance.

**Table 3:** Genetic conditions by morbidity level.

Morbidity Level	Gene	Disease	Clinical Picture
High morbidity	*CYP21A2*	Congenital adrenal hyperplasia due to 21-hydroxylase deficiency	Life-threatening adrenal crises in infancy if untreated (Classic salt-wasting type)
	*PKHD1*	AR polycystic kidney disease	Severe renal and hepatic involvement, pulmonary hypoplasia, and high neonatal mortality
	*SMN1*	Spinal muscular atrophy	Severe neuromuscular disease
	*COL7A1*	Dystrophic epidermolysis bullosa	Severe skin and mucosal involvement
	*TPP1*	Neuronal ceroid lipofuscinosis type 2	Fatal childhood neurodegenerative disease
	*HSD17B4*	Peroxisomal disorder	Severe encephalopathy and liver failure
	*DHCR7*	Smith–Lemli–Opitz syndrome	Multiple malformations and severe intellectual disability
	*SMPD1*	Niemann–Pick disease type A/B	Lysosomal storage disorder (type A often lethal)
	*EYS*	Retinitis pigmentosa	Progressive retinal degeneration leading to blindness
Moderate morbidity	*CFTR*	Cystic fibrosis	Affects lungs, pancreas, and reproductive system
	*PAH*	Phenylketonuria	Cognitive impairment if untreated; diet-controlled
	*EVC*	Ellis–van Creveld syndrome	Chondrodysplastic dwarfism with possible cardiac defects
	*ALDOB*	Hereditary fructose intolerance	Potentially severe but reversible with diet
	*SERPINA1*	Alpha-1 antitrypsin deficiency	Chronic condition affecting lungs and/or liver
	*SLC26A2*	Skeletal dysplasias	Variable severity (from lethal to moderate forms)
Low morbidity	*HFE*	Hereditary hemochromatosis	Low morbidity; manageable with phlebotomy and monitoring
	*BTD*	Biotinidase deficiency	Completely reversible
	*GJB2*	Nonsyndromic congenital hearing loss	Classically severe, but milder variants exist
	*LIFR*	Stüve–Wiedemann syndrome	Variable severity depending on mutation

**Table 4:** Genetic conditions by actionability.

Actionability	Gene	Disease	Actionable Measures
Medically actionable	*HFE*	Hereditary hemochromatosis	Preventable organ damage with phlebotomy and monitoring [9]
	*CFTR*	Cystic fibrosis	Targeted therapies (CFTR modulators), early pulmonary & nutritional management [10]
	*SMN1*	Spinal muscular atrophy	Disease-modifying therapies available (Nusinersen, Onasemnogene, Risdiplam) [11]
	*BTD*	Biotinidase deficiency	Completely preventable with biotin supplementation [12]
	*ALDOB*	Hereditary fructose intolerance	Disease prevented by dietary exclusion of fructose and KHK inhibition [13]
	*PAH*	Phenylketonuria	Diet ± pharmacologic therapy [14]
	*CYP21A2*	Congenital adrenal hyperplasia due to 21-hydroxylase deficiency	Glucocorticoid and mineralocorticoid replacement immediately after birth and lifelong for prevention of adrenal crisis [15]
	*GJB2*	Nonsyndromic congenital hearing loss	Early intervention (hearing aids, cochlear implants, speech therapy) [16]
	*SERPINA1*	Alpha-1 antitrypsin deficiency	Intravenous alpha-1 antitrypsin, COPD ^1^ therapies, Liver transplant [17]
	*TPP1*	CLN2 ^2^ disease	Enzyme replacement therapy slows neurodegeneration (Cerliponase alfa) [18]
Partially actionable	*SMPD1*	Niemann–Pick disease A/B	Enzyme replacement for type B [19] but type A largely non-actionable
	*HSD17B4*	Peroxisomal disorders	Supportive management only (no curative therapy) [20]
	*PKHD1*	AR polycystic kidney disease	Treatment of congenital hepatic fibrosis and portal hypertension, (dialysis) and kidney transplant [21]
	*SLC26A2*	Skeletal dysplasia	Orthopedic and supportive interventions(no molecular cure) [22]
	*COL7A1*	Dystrophic epidermolysis bullosa	Surgical pseudosyndactyly release, emerging gene therapies and novel skin grafts [23]
Limited clinical actionability	*DHCR7*	Smith–Lemli–Opitz syndrome	Management is Symptomatic/supportive [24,25,26,27]
	*EVC*	Ellis–van Creveld syndrome	
	*EYS*	Retinitis pigmentosa	
	*LIFR*	Stüve–Wiedemann syndrome	

^1^ Chronic Obstructive Pulmonary Disease; ^2^ Classic late-infantile neuronal ceroid lipofuscinosis.

**Table 5:** Origins and regional distribution of study participants in Romania.

Romanian County	Frequency	Percent	Valid Percent	Cumulative Percent
Arad	27	5.47	5.47	5.47
Bihor	2	0.41	0.41	5.87
Bistria-Nasaud	2	0.41	0.41	6.28
Caras-Severin	19	3.85	3.85	10.12
Dolj	2	0.41	0.41	10.53
Foreign	4	0.81	0.81	11.34
Gorj	9	1.82	1.82	13.16
Galati	1	0.20	0.20	13.36
Hunedoara	13	2.63	2.63	15.99
Ilfov	2	0.41	0.41	16.40
Iasi	5	1.01	1.01	17.41
Mehedinti	4	0.81	0.81	18.22
Maramures	2	0.41	0.41	18.62
Mures	3	0.61	0.61	19.23
Neamt	2	0.41	0.41	19.64
Not specified	2	0.41	0.41	20.04
Olt	2	0.41	0.41	20.45
Prahova	4	0.81	0.81	21.26
Sibiu	2	0.41	0.41	21.66
Satu Mare	2	0.41	0.41	22.07
Tulcea	1	0.20	0.20	22.27
Timis	383	77.53	77.53	99.80
Valcea	1	0.20	0.20	100.00
Missing	0	0.00		
Total	494	100.00		

## Data Availability

The data presented in this study are available on request from the corresponding authors.

## Supplementary Materials

The following supporting information can be downloaded at: https://www.mdpi.com/article/10.3390/ijms27083581/s1.

## Abbreviations

The following abbreviations are used in this manuscript:

ACOG	American College of Obstetricians and Gynecologists
AD	Autosomal Dominant
AMP	Association for Molecular Pathology
AR	Autosomal Recessive
bp	Base pairs
CA	California
CF	Cystic fibrosis
CFTR modulator	Cystic Fibrosis Transmembrane Conductance Regulator modulator
CLN2	Classic late-infantile neuronal ceroid lipofuscinosis
CNV	Copy Number Variant
COPD	Chronic Obstructive Pulmonary Disease
DNA	Deoxyribonucleic acid
ESHG	European Society of Human Genetics
ESHRE	European Society of Human Reproduction and Embryology
EURORDIS	Rare Diseases Europe
GRCh37	Genome Reference Consortium Human build 37
IVF	In Vitro Fertilization
NGS	Next-Generation Sequencing
PGT-M	Preimplantation genetic testing for monogenic disorders
PLP	Pathogenic or likely pathogenic
PCR	Polymerase Chain Reaction
RGCS	Reproductive genetic carrier screening
SMA	Spinal muscular atrophy
USA	United States of America
VUS	Variant of Uncertain Significance
WES	Whole-exome sequencing
