# Supplementary figures and images for: Reproductive Genetic Carrier Screening in Romania: A Couple-Based Study of Pathogenic Molecular Variants
Source: Int J Mol Sci. 2026 Apr 17;27(8):3581. doi: 10.3390/ijms27083581 (PMC13115674; doi:10.3390/ijms27083581)

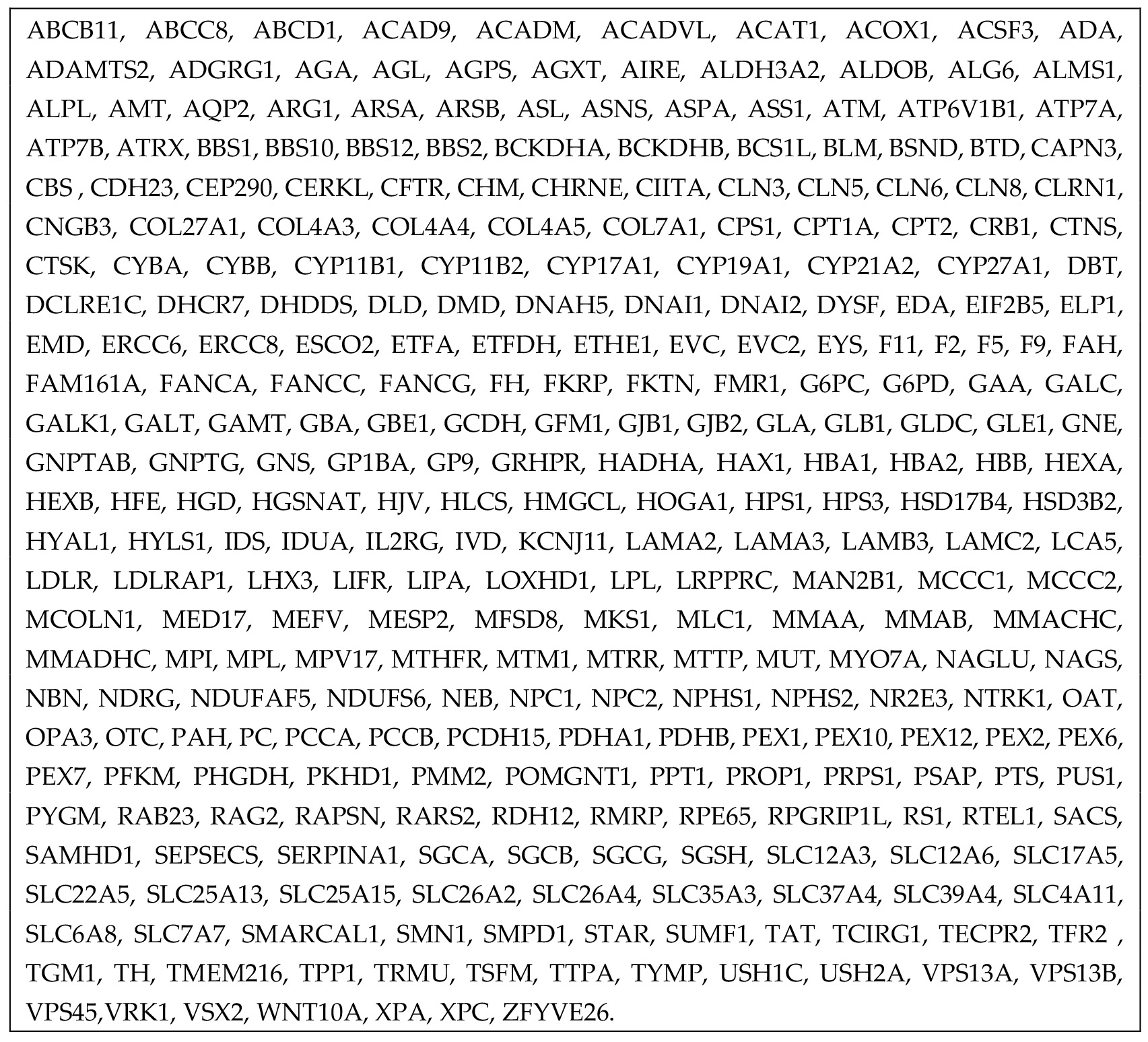

Supplement: Supplementary file 1 [file ijms-27-03581-s001.zip › Figure S1.jpg]
